# Stomatocytes and macrothrombocytopenia: A blood film for a rare disease

**DOI:** 10.1002/jha2.55

**Published:** 2020-07-04

**Authors:** Alessandro La Rosa, Erica Ricci, Francesca Scuderi, Concetta Micalizzi, Maja Di Rocco

**Affiliations:** ^1^ DINOGMI University of Genova Genoa Italy; ^2^ Unit of Rare Diseases IRCCS Istituto Giannina Gaslini Genoa Italy; ^3^ Haematology Unit IRCCS Istituto Giannina Gaslini Genoa Italy; ^4^ Laboratory of Hematology Department of Pediatric Hematology and Oncology IRCCS Istituto Giannina Gaslini Genoa Italy

A 15‐year‐old boy, native of Sudan from consanguineous parents, was evaluated for a 1‐year history of arthralgias, partially responsive to analgesic therapy, and the presence of stretch‐elastic consistency skin lesions on the extensor surfaces of joints. Basal blood tests showed mild normocytic anaemia and thrombocytopenia, hypergammaglobulinaemia, hyperferritinaemia and increased inflammatory index. Joints and bones radiological evaluation was normal. The skin lesion biopsy revealed extensive infiltrate of foamy histiocytes (CD68+; S100‐) compatible with xanthoma.

Blood tests were repeated and showed mild trilinear cytopenia (haemoglobin 119 g/L, white blood cells 3.4 × 10^9^/L, lymphocytes 17.3%, platelets 107 × 10^9^/L and mean platelet volume 15.3 fL [normal range (n.r.) 7–12 fL]), high inflammatory indexes, elevated levels of total and low‐density lipoprotein cholesterol (respectively, 386 mg/dL [n.r. 80‐180] and 308 mg/dL [n.r. 130‐160 mg/dL]) and low level of high‐density lipoprotein cholesterol (46 mg/dL (n.r. 55‐100 mg/dL). The blood film confirmed the presence of giant platelets with vacuolated cytoplasm and stomatocytes (Figure 1A,B); bone marrow aspiration did not show any abnormalities of megakaryocytes. In consideration of the clinical history and blood tests, the diagnosis of sitosterolaemia was suspected and confirmed by the presence of the homozygous pathogenetic variant *c.490C>T p.(Arg164)* of *ABCG8* gene; a cardiovascular complications work‐up shows initial thickening of the common carotids, compatible with premature atherosclerosis.

**FIGURE 1:**
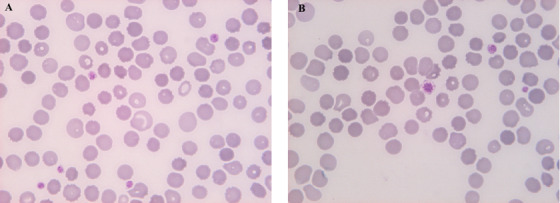
Patient's blood film: **A**, Stomatocytes, erythrocytes with a slit‐like central pallor, giving them the appearance of “kissing lips.” **B**, Macrothrombocytes, platelet larger than usual, with almost the same size of a red blood cell

Sitosterolaemia is an autosomal recessive disorder resulting from mutation of *ABCG5* or *ABCG8*, leading to excessive absorption and accumulation of plant sterols in plasma and tissues. The clinical spectrum is heterogeneous and patients may present with arthralgia/arthritis, xanthomas, premature atherosclerosis, or haematological signs such as haemolytic anaemia and macrothrombocytopenia. Correct diagnosis has become particularly important with the availability of specific treatment with ezetimibe associated with a low‐plant sterol diet that our patient promptly started. The aim of this case study is to emphasize that simple tests, such as blood films, sometimes allow to suspect rare diseases.

## AUTHOR CONTRIBUTIONS

Maja Di Rocco and Concetta Micalizzi were the clinicians primarily responsible for the patient's care; Francesca Scuderi prepared the blood films and took the patient's pictures; Alessandro La Rosa prepared the draft of the clinical picture; and Maja Di Rocco and Erica Ricci reviewed it. All the authors approved the final submitted version. Written consent to publish was obtained from the tutor of the patient.

## CONFLICT OF INTEREST

The authors declare that there is no conflict of interest.

